# 221 Controlling a Candida auris Outbreak in a Pediatric ICU: Impact of Genomic Surveillance, Disinfection, and Unit-Level Source Control

**DOI:** 10.1017/ash.2026.10445

**Published:** 2026-06-23

**Authors:** Joan Ivaska, Aarikha D’Souza, Morgan Baker, Steven Watson, Alice Hughes

**Affiliations:** 1 Banner Health

## Abstract

**Background:** Infection prevention (IP) workflows and staffing continue to be largely designed to provide retrospective surveillance with improvement strategies implemented after infections have occurred despite strong evidence that near real time feedback on clinical care and prevention bundles result in improved clinical care and prevention of infections. While some organizations have implemented centralized surveillance teams to manage chart abstraction for state and national reporting, few have moved toward a prospective surveillance model with their facility-based IP teams. Having implemented a centralized surveillance team in 2015, and an ambulatory specialty IP team in 2017 across a large multi-state healthcare system, a comprehensive IP model redesign was undertaken in 2025 to further transition to a prospective surveillance approach. **Methods:** Starting January 2025, a prospective surveillance program was launched, combining centralized surveillance, hospital-based generalists, and zone-based specialists in IP. Specialty teams were developed for surgical and procedural services, adult critical care, ambulatory and emergency medicine, and oncology, womens, and pediatric services. Facility-based generalists provide coverage for observation and general medical-surgical units. Staff were assigned roles based on preference and qualifications. About 85% of work hours shifted to direct clinical observation with real-time feedback and coaching of clinical teams. **Results:** Standardized electronic rounding tools were utilized by both generalist and specialist IPs. Bundle compliance opportunities and trends were displayed on an electronic dashboard and emailed weekly to department leaders. Departments with consistent performance below expectations had to submit action plans to improve performance. In 2025 compared with 2024, total number of IP rounding observations increased 36.8%, CLABSI prevention bundle compliance improved by 8.7% with a 79% increase in the number of lines observed each month, urinary catheter care being completed increased 7.7% with a 52% increase in catheters observed each month, and SSI prevention bundle compliance improved by 8% with a 117% increase in surgical procedure observations. Conclusion Implementing a prospective generalist-specialist model in infection prevention led to significantly more clinical care observations and steady improvements in prevention strategy compliance. Increased visibility and presence on the units resulted in IPs becoming trusted members of the clinical care team. Having a specialist IP role also resulted in improved recruiting of IPs with specific clinical experience and boosting their credibility with the clinical teams. Limitations of this study include the staggered implementation of specialist roles per quarter that may have slowed improvement in specific locations.

